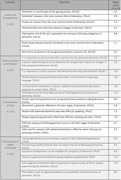# Assessing Alzheimer’s Disease Knowledge Among Caregivers Using the Alzheimer’s Disease Knowledge Scale

**DOI:** 10.1002/alz.090656

**Published:** 2025-01-09

**Authors:** Núria Guillén, Jordi Sarto, Diana Esteller, Andrea Val‐Guardiola, Guadalupe Fernandez‐Villullas, Ainoa Alberique, Gerard Piñol‐Ripoll, Mircea Balasa, Raquel Sánchez‐Valle, Neus Falgàs Martínez, Albert Lladó

**Affiliations:** ^1^ Alzheimer’s disease and other cognitive disorders Unit. Hospital Clínic de Barcelona; FRCB‐IDIBAPS; University of Barcelona, Barcelona Spain; ^2^ Hospital Universitari Santa Maria de Lleida, IRBLleida, Lleida Spain; ^3^ Hospital Clínic de Barcelona ‐ Fundació de Recerca Clínic Barcelona – IDIBAPS ‐ University of Barcelona, Barcelona, Catalonia Spain

## Abstract

**Background:**

Although Alzheimer’s disease (AD) is a common cause of dementia, whether patients and caregivers have notions of its risk factors, behavioral aspects or care considerations is unclear. Therefore, our study aims to evaluate caregiver’s knowledge of AD by using the Alzheimer’s Disease Knowledge Scale (ADKS).

**Method:**

The ADKS is a comprehensive 25‐item true/false assessment tool that explores the understanding of AD. Comprising four subscales (Causes and characteristics, Communication and behavior, Considerations of care, and Risk factors and health promotion), it provides a nuanced evaluation of knowledge levels.

In this prospective consecutive observational study conducted between February 2021 and December 2023, we recruited 132 participants with clinically diagnosed MCI/dementia. Their caregivers completed the Spanish version of the ADKS. Scores for the entire test, its subscales, and the individual questions were normalized on a scale from 0 to 10, to facilitate result interpretation.

**Result:**

The mean ADKS test score was 21.7 [SD 0.7], equivalent to 4.3/10 [SD 1.4]. Most of the subscales scored below 5, indicating a poor familiarity with aspects related to “Risk factors and health promotion” (4.3/10 [SD 1.8]), “Causes and characteristics” (4.2/10 [SD 1.9]), and “Communication and behavior” (2.0/10 [SD 1.6]) (Table 1). The only subscale with an average score over 5 was “Considerations of care,” (6.7/10 [SD 2.3]).

**Conclusion:**

The findings underscore the limited knowledge of AD‐related aspects among caregivers of individuals suffering from cognitive impairment. It highlights the need to implement educational interventions in both the general population and diagnosed patients and their support networks.